# Morphologic Features of Extrahepatic Manifestations of Hepatitis C Virus Infection

**DOI:** 10.1155/2012/740138

**Published:** 2012-08-05

**Authors:** Huaibin M. Ko, Juan C. Hernandez-Prera, Hongfa Zhu, Steven H. Dikman, Harleen K. Sidhu, Stephen C. Ward, Swan N. Thung

**Affiliations:** The Lillian and Henry M. Stratton-Hans Popper Department of Pathology, Mount Sinai School of Medicine, New York, NY 10029, USA

## Abstract

Cirrhosis and hepatocellular carcinoma are the prototypic complications of chronic hepatitis C virus infection in the liver. However, hepatitis C virus also affects a variety of other organs that may lead to significant morbidity and mortality. Extrahepatic manifestations of hepatitis C infection include a multitude of disease processes affecting the small vessels, skin, kidneys, salivary gland, eyes, thyroid, and immunologic system. The majority of these conditions are thought to be immune mediated. The most documented of these entities is mixed cryoglobulinemia. Morphologically, immune complex depositions can be identified in small vessels and glomerular capillary walls, leading to leukoclastic vasculitis in the skin and membranoproliferative glomerulonephritis in the kidney. Other HCV-associated entities include porphyria cutanea tarda, lichen planus, necrolytic acral erythema, membranous glomerulonephritis, diabetic nephropathy, B-cell non-Hodgkin lymphomas, insulin resistance, sialadenitis, sicca syndrome, and autoimmune thyroiditis. This paper highlights the histomorphologic features of these processes, which are typically characterized by chronic inflammation, immune complex deposition, and immunoproliferative disease in the affected organ.

## 1. Introduction

Hepatitis C is a disease that affects approximately 170 million people worldwide, with a prevalence in the United States of approximately 2% of the adult population [1]. Chronic hepatitis C occurs in 80% of these cases and can lead to cirrhosis and hepatocellular carcinoma [2]. Extrahepatic manifestations (EHMs) of hepatitis C virus (HCV) infection were first reported in the early 1990s [3] and can affect a variety of organ systems with significant morbidity and mortality. Forty to 75% of patients with chronic HCV infection exhibit at least one clinical EHM [4, 5].

HCV infection is generally characterized by an indolent clinical course that is influenced by a variety of host, viral, and environmental factors [6]. While HCV may infect other cells outside of the liver, most EHMs are thought to be secondary to the host immune response to the viral infection and not a direct viral cytopathic effect [7, 8]. The natural history of HCV infection and its association with EHMs is only partially understood. Some EHMs, such as mixed cryoglobulinemia, have been strongly associated with hepatitis C both clinically and pathologically, while other EHMs may be linked to HCV based on higher prevalence, response to antiviral treatment, or anecdotal observation.

## 2. Mechanisms

While direct infection of extrahepatic tissue cells by HCV has been documented, the majority of EHMs are thought to be secondary to immune-mediated mechanisms, either lymphoproliferative or autoimmune in nature.

HCV infection results in upregulation of the humoral immune system in patients with chronic disease, which leads to increases in monoclonal and polyclonal autoantibodies via chronic antigenic stimulation [7]. It has been postulated that anti-HCV-IgG and HCV lipoprotein complexes may act as B-cell superantigens inducing the synthesis of non-HCV reactive IgM with rheumatoid factor-like activity [9]. These autoantibodies, in turn, form immune complexes, which circulate through the body and are deposited in small to medium blood vessels, resulting in complement activation and extrahepatic injury [7–9].

## 3. Mixed Cryoglobulinemia

HCV is associated with essential mixed cryoglobulinemia (MC), also known as type II cryoglobulinemia. MC is the most documented extrahepatic manifestation of chronic HCV infection and is found in more than half the patients [10–13]. Of these 10% are symptomatic [13, 14].

Cryoglobulins are circulating immunoglobulins that precipitate with cold temperature and resolubilize when warmed. In type II cryoglobulinemia, the cryoglobulins are composed of two or more classes of different immunoglobulins of which one is a monoclonal IgM component with rheumatoid factor-like activity [15]. Expansion of rheumatoid factor synthetizing B cells represents the biological hallmark of MC [16]. Many organs including the skin, gastrointestinal tract, and kidney may be involved. The classic triad of symptoms in patients with HCV-associated MC is palpable purpura, weakness, and arthralgia.

### 3.1. Palpable Purpura/Leukoclastic Vasculitis

**Figure 1:**
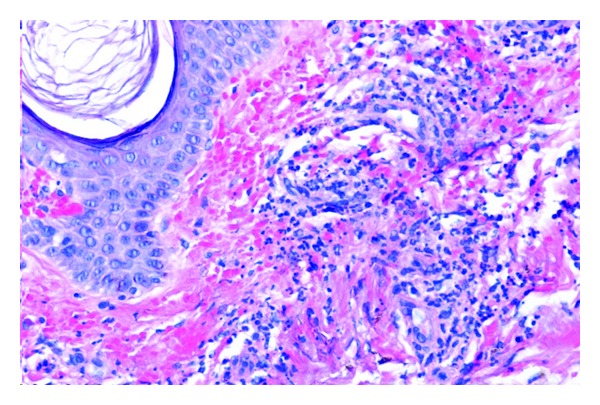
Leukocytoclastic vasculitis: predominantly lymphocytic mixed inflammatory infiltrate involving small vessels in the dermis (hematoxylin-eosin, original magnification ×200).

**Figure 2:**
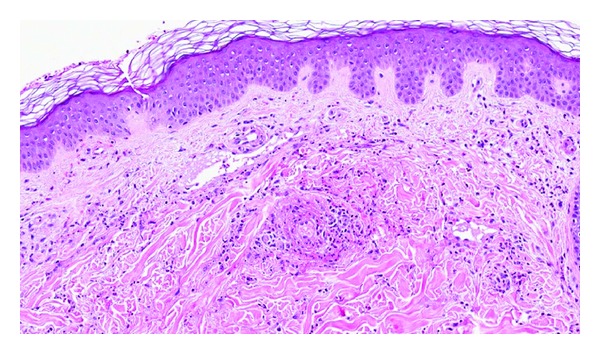
Leukocytoclastic vasculitis: fibrinoid necrosis of dermal vessels (hematoxylin-eosin, original magnification ×100) (photo courtesy of Dr. Rajendra Singh).

Cutaneous vasculitis of HCV-related MC, resulting in palpable purpura, is reported in 24–30% of cryoglobulin positive patients [4, 17]. It is secondary to small and/or medium vessel vasculitis with deposition of immune complexes in the small- and medium-sized dermal vessels [17]. It occurs intermittently, preferentially during the winter months, and is nonpruritic. It characteristically begins with involvement of the lower limbs and moves cranially toward the abdomen, less frequently involving the trunk and upper limbs. The face is always spared. The purpura is papular or petechial and persists for 3–10 days with residual brown pigmentation. In addition, Raynaud syndrome and acrocyanosis are found in 25–34% of patients [18]. Cutaneous biopsy shows a nonspecific mixed inflammatory infiltrate (leukocytoclastic vasculitis) involving small vessels (Figure 1). Mononuclear cells may be seen within the walls of the vessels, and, in some cases, endovascular thrombi and fibrinoid necrosis of the arteriolar walls may be seen (Figure 2).

### 3.2. Membranoproliferative Glomerulonephritis

**Figure 3:**
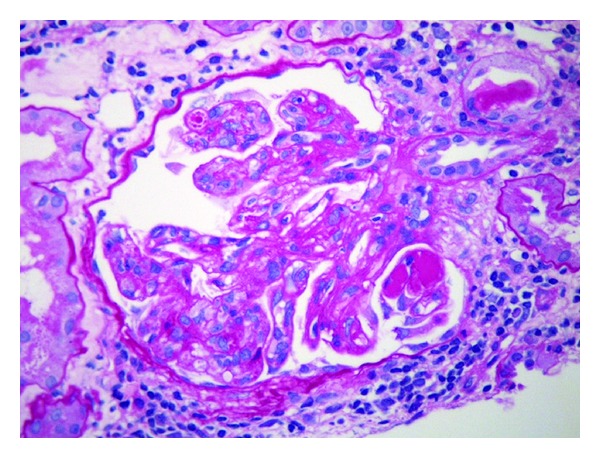
Membranoproliferative glomerulonephritis: PAS-positive “hyaline thrombi” seen within the capillary lumina (PAS, original magnification ×400).

Glomerulonephritis (GN), specifically, type I membranoproliferative glomerulonephritis (MPGN) is a common presentation of type II cryoglobulinemia in patients with chronic HCV infection. Patients may present with proteinuria and nephrotic syndrome [19–21]. On biopsy, a type I membranoproliferative glomerulonephritis is seen, sometimes with pronounced lobulation of the glomeruli [22]. There may be massive infiltration of the glomeruli by monocytes as well as diffuse thickening of the glomerular capillary wall [23]. Periodic acid-Schiff (PAS) positive “hyaline thrombi” can be seen within the capillary lumina (Figure 3). The light microscopy appearance may also appear as type III MPGN, acute exudative and proliferative glomerulonephritis. MPGN may be indistinguishable from allograft glomerulopathy in transplant patients [24]. As in other organs affected by mixed cryoglobulinemia, leukoclastic vasculitis can be seen in the kidney.

**Figure 4:**
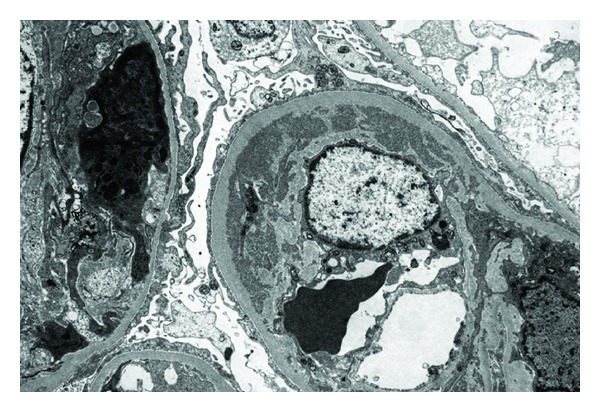
Membranoproliferative glomerulonephritis: endocapillary proliferation with extensive subendothelial deposits along the glomerular capillary walls. Mesangial deposits are present as well (electron microscopy).

**Figure 5:**
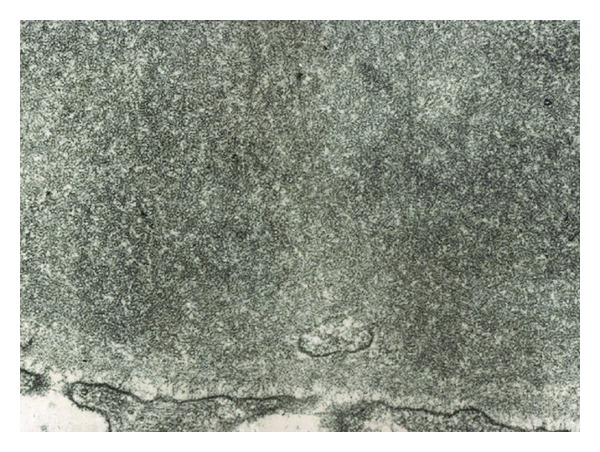
Membranoproliferative glomerulonephritis: subendothelial electron dense deposits in a patient with mixed essential cryoglobulinemia showing microtubular architecture. Microtubules measure approximately 30 nm in diameter (electron microscopy).

On immunofluorescence (IF) studies, coarsely granular deposits of IgG, IgM, and C3 are visualized in the capillary wall. On occasion, large intraglomerular deposits of C3 and other immunoreactants are seen forming “thrombi” within the glomerular capillaries [22]. Morphologically, the location and presence of the deposits is best seen on electron microscopy (EM). EM shows dense, immune-type mesangial and subendothelial deposits along the glomerular capillary walls (Figure 4). At high magnification, the cryoglobulin deposits often appear as organized tubular, cylindrical, or crystalloid deposits (Figure 5) [25]. Intramembranous and subepithelial deposits may be seen rarely, in addition to the subendothelial deposits [23, 25]. EM is useful in determining the presence and site of the deposits, which may be difficult to determine by IF [26].

## 4. Skin (Not Associated with Mixed Cryoglobulinemia)

### 4.1. Porphyria Cutanea Tarda

**Figure 6:**
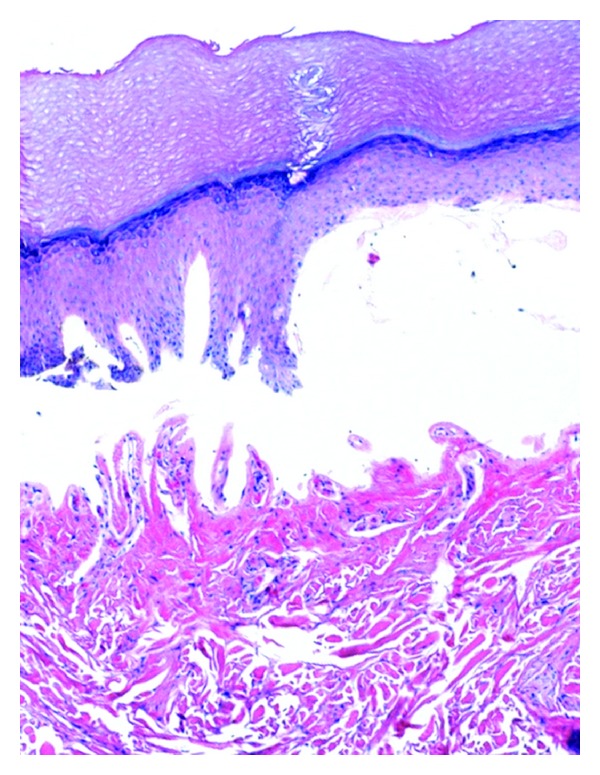
Porphyria cutanea tarda: subepidermal bulla and festooning of the dermal papilla are prominent. There is no significant inflammatory infiltrate (hematoxylin-eosin, original magnification ×100).

The prevalence of HCV infection in patients with porphyria cutanea tarda (PCT) varies according to region [9, 27]. The prevalence is higher in southern Europe (65%–91%) compared to northern Europe (8–17%). In Australia and New Zealand, the prevalence is about 20%; while, in the United States, the prevalence is reported to be 50–75% [27]. PCT results from decreased activity of the uroporphyrinogen decarboxylase enzyme; however, the mechanism that links this phenomena to chronic HCV infection is unknown. In most cases, HCV exposure and liver dysfunction precede the onset of PCT, suggesting that the HCV infection may uncover an existing porphyrin metabolism defect in susceptible patients. Histologically, PCT is characterized by cell poor subepidermal bulla with increased hyaline material in the vessel walls and basement membrane. The hyaline material is reactive in PAS-diastase-resistant staining [9]. The dermal papillae are rigid with festooning (Figure 6).

### 4.2. Lichen Planus

**Figure 7:**
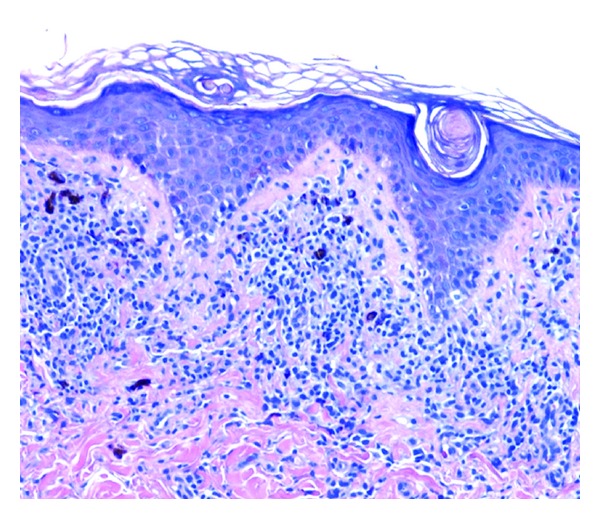
Lichen planus: there is a band-like infiltrate of lymphocytes at the epidermal-dermal junction with damage to the basal cell layer and pigment incontinence. The epidermis has a saw-toothed appearance (hematoxylin-eosin, original magnification ×200).

Lichen planus (LP) is a relatively common inflammatory skin disease in the general population and is thought to be related to autoimmunity [28]. The relationship between HCV infection and LP is controversial; however, literature analysis has found that, in most studies, the proportion of HCV-positive patients is higher in the LP group compared to the general population with the prevalence of HCV ranging from 16% to 55% and 1-2%, respectively [5, 29–31]. HCV-related LP lesions are similar to those of classic LP with the exception of oral involvement, which also occurs in the majority of HCV-related LP. Histologically, LP is characterized by band-like, subepidermal, lymphohistiocytic infiltrate with interface change, “sawtooth” rete ridges, and pigmentary continence (Figure 7) [9].

### 4.3. Necrolytic Acral Erythema

**Figure 8:**
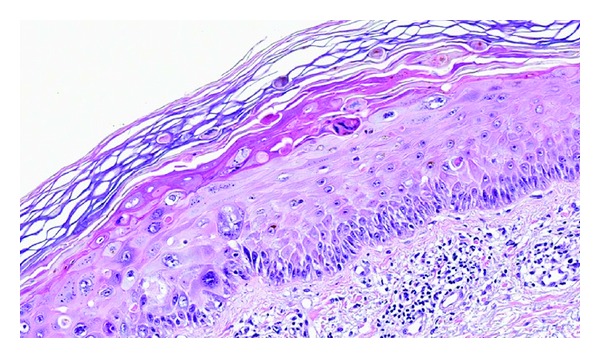
Necrolytic acral erythema: epidermal pallor in the stratum corneum, hyperkeratosis, dyskeratotic keratinocytes, spongiosis, and a superficial perivascular mixed inflammatory infiltrate in the dermis are seen (hematoxylin-eosin, original magnification ×200) (photo courtesy of Dr. Rajendra Singh).

Since its initial description in 1996, necrolytic acral erythema has been described as a dermatosis, which is almost exclusively associated with HCV infection [32]. It is characterized by pruritic, symmetric, well-demarcated, hyperkeratotic, erythematous-to-violaceous, lichenified plaques with a rim of dusky erythema on the dorsal aspects of the feet and extending to the toes. Disease remission has been described after oral zinc administration. Thus, it is thought that zinc dysregulation, which can occur in hepatitis C, is related to the pathogenesis of these lesions [33]. Morphologic features include a nonspecific psoriasiform pattern, acanthosis, papillomatosis, and hyper- and parakeratosis, with necrotic keratinocytes in the superficial epidermis. There is a superficial, perivascular inflammatory infiltrate comprised predominantly of lymphocytes. Some lymphocytes extend to a hyperplastic epidermis where there is spongiosis and foci of sharply demarcated parakeratosis (Figure 8) [34].

## 5. Kidney (Not Associated with Mixed Cryoglobulinemia)

### 5.1. Membranous Glomerulonephritis

Membranous GN may also occur in the setting of chronic HCV infection [19, 22]. In contrast to patients with HCV and MPGN, there is little evidence linking MGN to cryoglobulinemia. Patients with HCV and MGN do not appear to demonstrate cryoglobulinemia or rheumatoid factor [35], and hypocomplementemia is rarely found [36, 37]. Typically, early-stage MGN is seen, with no evidence of endocapillary proliferation [22]. Focal segmental glomerulosclerosis may be present [36]. IF studies show diffuse glomerular basement membrane granular deposits of IgG [37]. Membranous GN appears as subepithelial electron dense deposits on EM [36, 37].

### 5.2. Diabetic Nephropathy

Chronic infection with HCV is associated with insulin resistance (see the following). The gross and microscopic features of HCV-associated diabetic nephropathy are the same as diabetic nephropathy not associated with HCV. The kidneys may be enlarged in the early stages of disease due to hyperfiltration and hypertrophy of the glomeruli within the cortex. As the disease progresses, the kidney becomes scarred, with loss of nephrons, and decreases in size. However, end-stage disease does not commonly show gross contraction [38]. The corticomedullary junction arteries may be prominent due to arteriosclerosis, and the main renal artery may show atherosclerosis [22].

**Figure 9:**
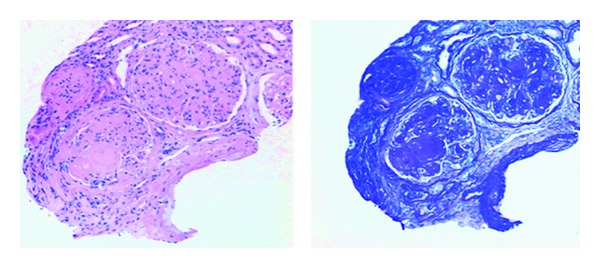
Diabetic Nephropathy: extensive mesangial expansion is seen, with rounded acellular mesangial nodules (Kimmelstiel-Wilson nodules) (hematoxylin-eosin and PAS, original magnification ×400).

The microscopic features include diffuse mesangial sclerosis with thickening of glomerular capillary walls and thickening of the tubular basement membranes. Nodular lesions, characterized as eosinophilic material within the mesangium first described by Kimmelstiel and Wilson in 1936, may be seen (Figure 9) [39]. Microaneurysms of glomerular capillary loops may precede the development of large nodules [22, 40]. On IF, linear staining along the glomerular capillary walls with IgG is seen [40, 41].

Exudative lesions, otherwise known as hyalinosis lesions or fibrous caps, are seen in 60% of diabetic kidneys and may be secondary to ischemia due to atherosclerosis [40]. These lesions consist of PAS-positive eosinophilic material that accumulates between endothelial cells and the glomerular basement membrane of the capillary loops, eventually filling the lumen of the capillaries [42, 43]. Hyalinosis lesions stain brightly on IF for IgM and C3 [41]. Hyalinosis lesions may also contain fibrinogen, lipoprotein, complement, *β*-lipoprotein, and small amounts of IgG [41]. “Capsular drop” is a lesion that stains similarly to hyalinosis lesions in the kidney. Capsular drop is identified as a round accumulation of eosinophilic material between the basement membrane and the parietal epithelial cells of Bowman capsule [43]. Adhesions between the glomerular lobule and Bowman capsule may be observed [22].

Other nonspecific findings that are associated with diabetic renal disease include hyaline arteriolosclerosis, interstitial fibrosis, chronic inflammatory infiltrates, and obsolete glomeruli [22].

## 6. Hematologic

### 6.1. B-Cell Non-Hodgkin Lymphoma

HCV is the stimulus not only for the apparent benign lymphoproliferative process underlying a wide spectrum of clinical features but also for the progression to frank lymphoid malignancy in a subgroup of patient [44]. Associations between chronic HCV infection and lymphoproliferative disorders have been described [45]. In patients with B-cell non-Hodgkin lymphoma (NHL), up to 13% have the HCV antibody [46, 47]. In addition, approximately 10% of patients with type II cryoglobulinemia associated with HCV developed NHL over a 10-year follow-up period [17]. It is believed that HCV E2 antigen binding to host CD81 receptors leads to B-cell proliferation, which may result in lymphoma [46, 48]. Regression of low-grade NHL has been observed in association with HCV therapy [49, 50]; however, high-grade HCV-associated lymphoma requires chemotherapy.

The majority of HCV-associated lymphomas are extranodal and located in the liver (primary hepatic lymphoma) and salivary glands [49, 50]. The bone marrow and spleen may also be involved [51, 52].

Morphologically, HCV-associated lymphomas represent a variety of histological subtypes. Marginal zone, lymphoplasmacytic, and diffuse large B-cell lymphomas are the most common histotypes associated with HCV [53, 54]. HCV has also been associated with follicular lymphoma [55, 56] as well as mucosa-associated lymphoid tissue (MALT) lymphoma, and mantle cell lymphoma [54].

**Figure 10:**
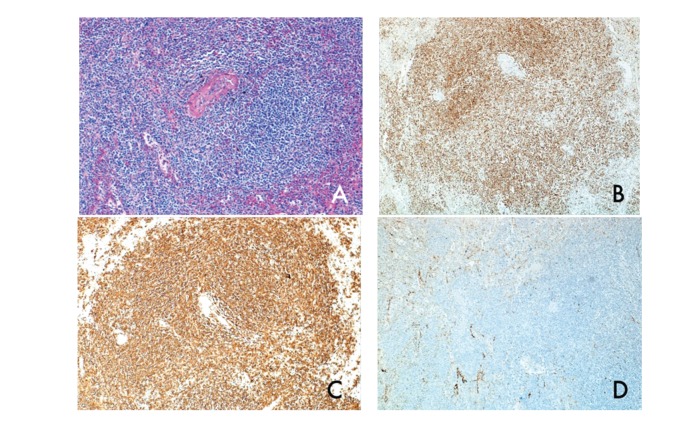
Marginal zone lymphoma of the spleen: (A) there is effacement of splenic architecture by sheets of monotonous small-to-medium size lymphocytes (hematoxylin-eosin, original magnification ×200). Immunohistochemical stains show that the lymphocytes are positive for BCL2 (B) and CD20 (C), and negative for CD10 (D) (immunoperoxidase, original magnifications ×200 ((A) through (D))).

Overall, marginal zone lymphoma appears to be the most frequently encountered low-grade B-cell lymphoma in HCV patients [52]. HCV infection is documented in approximately 35% of patients with nongastric B-cell marginal zone lymphoma [53]. Splenic marginal zone lymphoma, in particular has a high prevalence of HCV infection and is often associated with type II cryoglobulinemia [51, 53, 57]. Morphologically, a central zone of small round lymphocytes surrounds the germinal centers, commonly replacing the reactive germinal centers in the splenic white pulp. The red pulp is infiltrated with small lymphocytes and ill-defined nodules of larger cells (Figure 10) [58]. The tumor cells stain positively for CD20, CD79a, and BCL2 in the majority of cases. They are negative for CD5, CD10, CD23, and annexin A1 [58].

HCV-associated lymphoplasmacytic lymphoma (LPL) has been associated with type II cryoglobulinemia in some studies and may be related to geographic location [58]. Morphologically, LPL presents as a relatively monotonous proliferation of small lymphocytes, plasma cells, and plasmacytoid lymphocytes. Dutcher bodies (plasma cells with PAS+ intranuclear inclusions) and mast cells may be seen [58].

Primary hepatic diffuse large B-cell lymphoma (DLBCL) is also associated with HCV. DLBCL may present on histology as large lymphoid cells with vacuolated nuclei in a diffuse infiltrating pattern intermingled with small lymphoid cells. The large cells typically stain positively for CD20, CD10, and CD25 [59]. On occasion, NHL may be seen concurrently with hepatocellular carcinoma [60].

## 7. Autoimmune/Inflammatory

### 7.1. Type 2 Diabetes Mellitus

Chronic infection with HCV is associated with insulin resistance, metabolic syndrome, and type 2 diabetes [61]. The prevalence of type 2 diabetes has been reported to be 14–50% in patients with chronic HCV infection [62]. HCV-associated diabetes is characterized by insulin resistance and does not appear to be associated with antibodies directed towards the beta cells in the islets of Langerhans [63]. The mechanism for insulin resistance is unclear, but it is thought to be secondary to viral induced adipocytokine release or HCV viral proteins directly interfering with inflammatory or muscle insulin signaling pathways [64]. HCV-related type 2 diabetes mellitus occurs in association with hepatic steatosis, insulin resistance, and high levels of both tumor-necrosis factor and CXCL10 [65].

### 7.2. Sialadenitis/Sicca Syndrome

**Figure 11:**
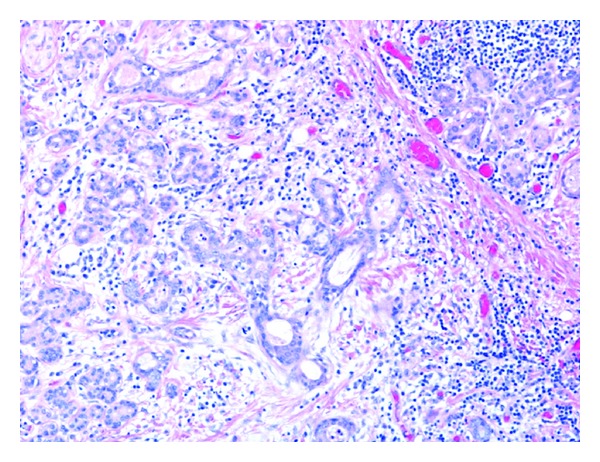
Sialadenitis: there is extensive lymphoid infiltrate with interstitial fibrosis and acinar atrophy (hematoxylin-eosin, original magnification ×100).

The association between sialadenitis and HCV infection was first postulated in 1992, and the reported occurrence of HCV-related sicca syndrome ranges from 4 to 57% of chronic HCV patients [4, 61, 66, 67]. The large range may be related to differences in diagnostic criteria [61]. The mechanism by which HCV results in sicca syndrome is not well established. The virus has not been shown to directly infect salivary gland tissue [68], and it is likely that HCV-related sicca syndrome is the product of host immune-mediated mechanism, rather than direct viral effect [69]. Patients may present with oral or ocular dryness. Histologic examinations of salivary gland biopsies in HCV-infected patients show pericapillary and nonpericanalary lymphocytic infiltration. The glandular canals are typically spared (Figure 11) [61].

### 7.3. Autoimmune Thyroiditis

Autoimmune thyroid disease is commonly associated with HCV. Hypothyroidism is seen in 3.5–13% of patients with chronic HCV [70, 71]. Patients generally present with the most common cases of autoimmune thyroid disease: Graves's disease (GD) and Hashimoto's thyroiditis (HD). The pathogenesis of HCV-related autoimmune thyroid disease is unknown. Two hypotheses have been proposed: primary viral cytopathic effect and secondary induced autoimmunity [72]. Interferon therapy may also induce antithyroid antibodies or uncover underlying Hashimoto's thyroiditis or Graves's disease, which can be refractory to discontinuation of therapy [71, 73].

**Figure 12:**
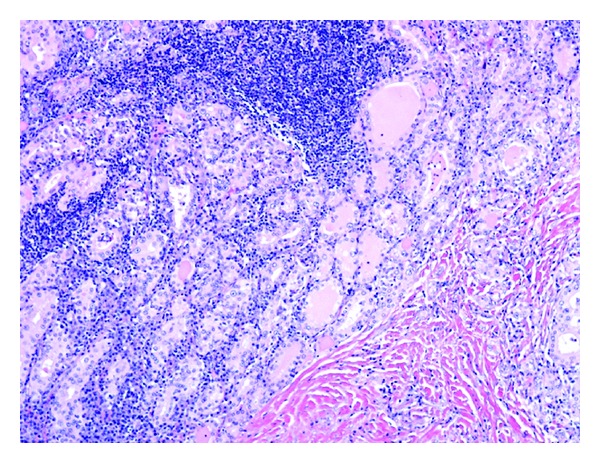
Hashimoto thyroiditis: there is extensive lymphocytic infiltrate with germinal center formation. Follicular cells are slightly enlarged with partial nuclear clearing (hematoxylin-eosin, original magnification ×100).

In the early stages of Hashimoto's thyroiditis, the thyroid is firm, symmetrically enlarged, and has a tan-yellow appearance corresponding to lymphoid tissue on gross examination. The gland may become atrophic in end-stage disease. Histological findings include small, atrophic thyroid follicles with lymphoplasmacytic infiltration and well-developed germinal centers (Figure 12) [26]. The lymphocytic infiltrate is composed of mixed T and B cells in an even ratio [26].

**Figure 13:**
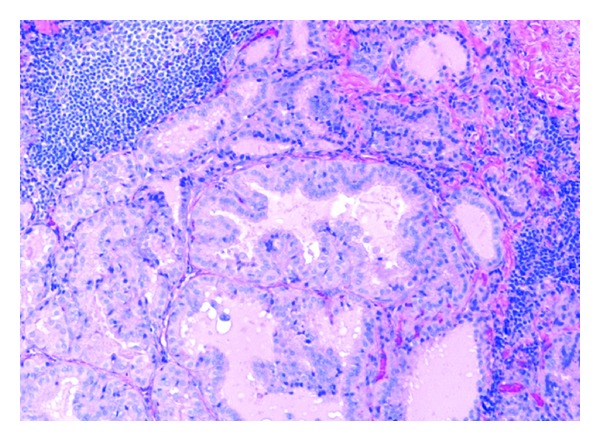
Graves disease: there is follicular hyperplasia with intracellular colloid droplets, cell scalloping, a reduction in follicular colloid, and a multifocal lymphocytic infiltrate (hematoxylin-eosin, original magnification ×200).

In Graves's disease, the thyroid has marked vascularity and is diffusely enlarged. Histologic examination shows prominent vascular congestion, follicular hyperplasia, and papillary hyperplasia. The follicular cells appear columnar with enlarged nuclei that may demonstrate nuclear clearing, mimicking papillary carcinoma (Figure 13) [26]. Reactive lymphocytes are found in the stroma [74].

## 8. Conclusion

Chronic hepatitis C virus infection is associated with multiple extrahepatic manifestations (EHMs) affecting various organs in the body. While there is some evidence that the virus may play a direct role in HCV-related B-cell lymphomas via direct HCV antigen stimulation of B-cells, most EHMs are generally believed to be secondary to the host immune response to the virus.

In some conditions, the histopathologic changes of EHM are related to circulating immune complexes such as type II cryoglobulinemia, and their subsequent deposition in the small vessels and glomerular capillary walls, leading to leukoclastic vasculitis in the skin and membranoproliferative glomerulonephritis in the kidney.

Other HCV-associated entities like sialadenitis, sicca syndrome, lichen planus, and autoimmune thyroiditis, while not associated with cryoglobulinemia, appear to be secondary to autoimmune processes resulting in chronic inflammatory infiltrates.

In porphyria cutanea tarda, the disease process is thought not to be related to host immune response to HCV, but rather to HCV-associated liver dysfunction.

The role of the virus in insulin resistance in HCV-associated diabetes is unclear, but it is thought to be secondary to either viral induced inflammation or direct interference of the virus on muscle insulin signaling.

In summary, chronic HCV infection may result in a multitude of disease processes affecting the small vessels, skin, kidneys, salivary glands, eyes, thyroid, and immunologic system. The sequelae of extrahepatic HCV infection are seen histomorphologically as chronic inflammation, immune complex deposition, and immunoproliferative disease in the affected organs.
